# Supplementary material for: Gain-of-function genetic screening identifies the antiviral function of TMEM120A via STING activation
Source: Nat Commun. 2022 Jan 10;13:105. doi: 10.1038/s41467-021-27670-1 (PMC8748537; doi:10.1038/s41467-021-27670-1)
Supplement: Supplementary file 1 — Supplementary Information [file 41467_2021_27670_MOESM1_ESM.pdf]

Supplementary Information

Gain-of-function genetic screening identifies the antiviral function of TMEM120A via STING activation

Authors:

Shuo Li[1,2,#], Nianchao Qian[1,2,#], Chao Jiang[1,2], Wenhong Zu[1], Anthony Liang[3], Mamie Li[3], Stephen J. Elledge[3] and Xu Tan[1,2*]

Affiliations:

1. Beijing Advanced Innovation Center for Structural Biology, Beijing Frontier Research Center for Biological Structure, MOE Key Laboratory of Bioorganic Phosphorus Chemistry & Chemical Biology, School of Pharmaceutical Sciences, Tsinghua University, Beijing 100084, China

2. Tsinghua-Peking Center for Life Sciences, Beijing, 100084, China

3. Division of Genetics, Brigham and Women's Hospital, Howard Hughes Medical Institute, Department of Genetics, Harvard Medical School, Boston, MA, 02120， USA.

[#]. These authors contributed equally to this work.

***Correspondence should be addressed to:** Xu Tan (Email: xutan@tsinghua.edu.cn)

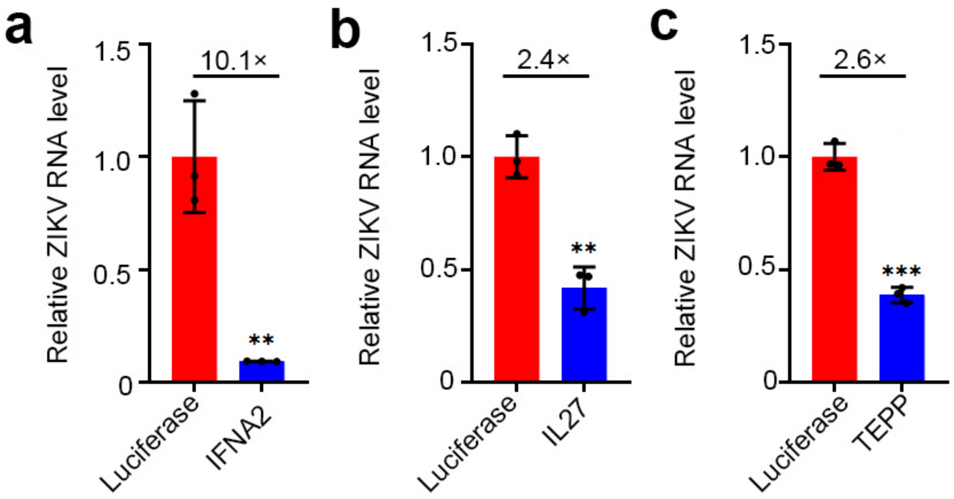

Supplementary Figure 1. Validation of IFNA2, IL27, TEPP for anti-ZIKV function in U87MG cells.

U87MG cells stably expressing luciferase or IFNA2 (a), IL27 (b), TEPP (c) were infected with ZIKV at an MOI of 0.1 for 2 days. Cells were then harvested for RT-qPCR. Cellular ZIKV RNA level was normalized to the internal control GAPDH. Data represent the mean ± SEM (n=3 independent experiments). **: P<0.01, ***: P<0.001 (unpaired, two-sided Student's t-test). Exact P values and statistical parameters are provided in Source Data File.

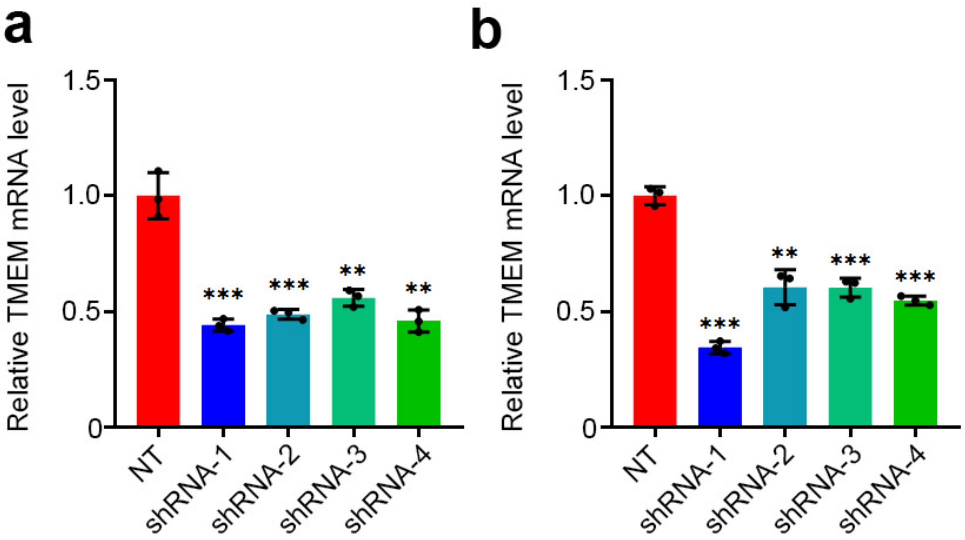

Supplementary Figure 2. TMEM120A knockdown efficiency.

Huh7 (a) and U87MG (b) cells stably expressing NT shRNA or TMEM120A shRNAs (four independent shRNAs: shRNA-1, shRNA-2, shRNA-3, shRNA-4) were infected with ZIKV at an MOI of 0.1 for 48h. Cells were then harvested for RT-qPCR. TMEM120A mRNA level was normalized to the internal control GAPDH. Data represent the mean ± SEM (n=3 independent experiments). **: $P<0.01$, ***: $P<0.001$ (one-way ANOVA and Dunnett's test). Exact *P* values and statistical parameters are provided in Source Data File.

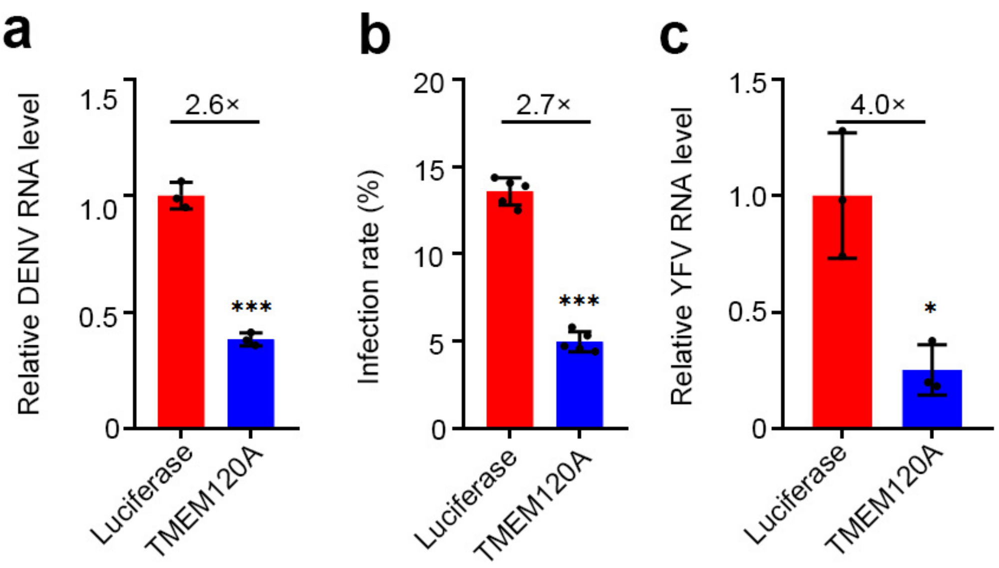

Supplementary Figure 3. TMEM120A expression inhibits DENV and YFV infection in U87MG cells.

(a) TMEM120A overexpression significantly reduced ZIKV RNA level in U87MG cells. U87MG cells stably expressing luciferase or TMEM120A were infected with DENV at an MOI of 0.1 for 48h. Cells were then harvested for RT-qPCR.

(b) TMEM120A overexpression inhibited DENV infection rate in U87MG cells. U87MG cells stably expressing luciferase or TMEM120A were infected with DENV at an MOI of 0.2 for 48h. Cells were then fixed, permeabilized for immunostaining of DENV envelope protein. The infection rate was measured by high content scanning.

(c) TMEM120A overexpression significantly reduced YFV RNA level in U87MG cells. U87MG cells stably expressing luciferase or TMEM120A were infected with YFV at an MOI of 0.1 for 48h. Cells were then harvested for RT-qPCR.

Cellular DENV RNA level was normalized to the internal control GAPDH. RT-qPCR data in (a, c) represent the mean ± SEM (n=3 independent experiments). ZIKV infection rate in (b) represent the mean ± SEM (n=5 independent experiments). *: $P<0.05$, ***: $P<0.001$ (unpaired, two-sided Student's *t*-test). Exact *P* values and statistical parameters are provided in Source Data File.

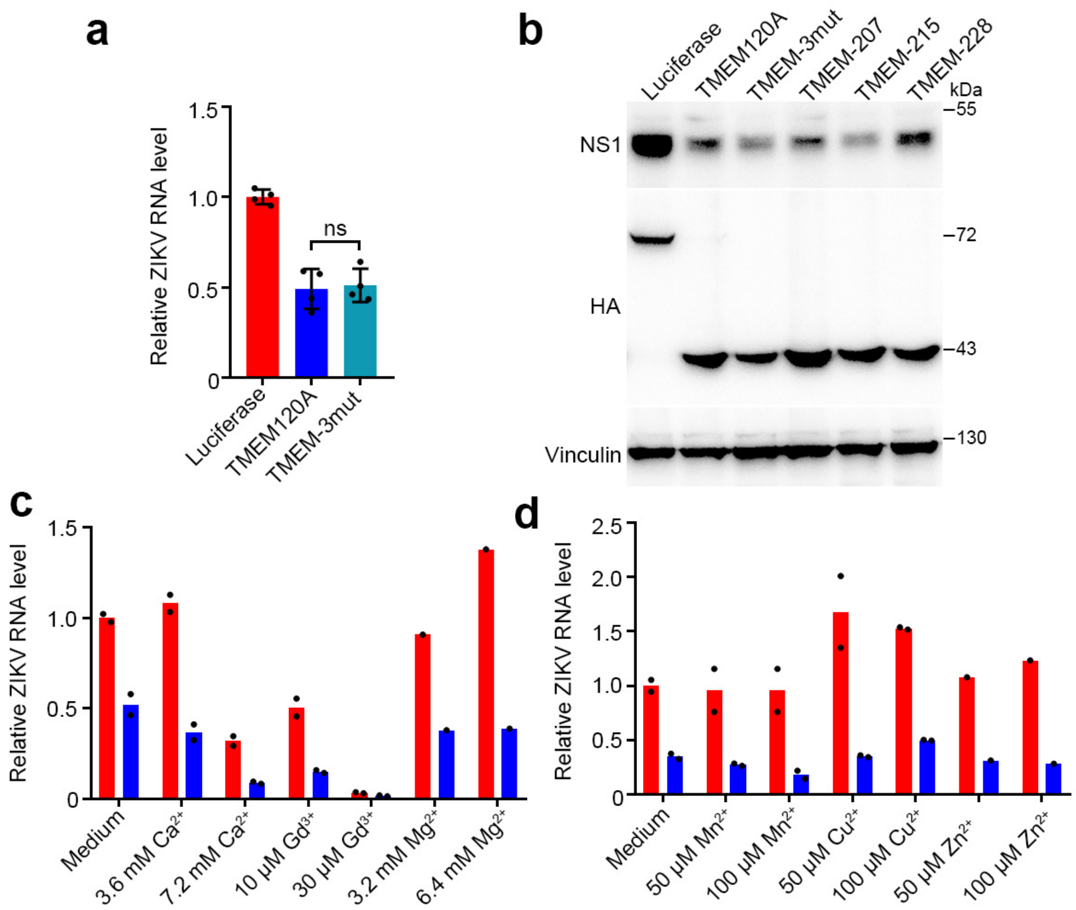

Supplementary Figure 4. The anti-viral function of TMEM120A is independent on its ion channel function. (a, b) TMEM120A mutations did not rescue ZIKV RNA level (a) or NS1 protein level (b) in U87MG cells. Alanine mutations were introduced in TMEM120A aa 207, 215, 228 or all the 3 sites (TMEM120A M207A, M215A, M228A and 3mut) which have been reported to be the ion-conducting part responsible for mechanically-evoked currents. U87MG stably expressing HA- FLAG tagged luciferase, TMEM120A or TMEM120A mutations were infected with ZIKV at an MOI of 0.1 for 48h. Cells were then harvested for RT-qPCR and immunoblotting (NS1 antibody).

(c, d) Ion treatments did not abolish the anti-viral activity of TMEM120A in U87MG cells. U87MG stably expressing luciferase or TMEM120A were treated with the indicated ions and infected with ZIKV at an MOI of 0.1 for 48h. Cells were then harvested for RT-qPCR.

Cellular ZIKV RNA level was normalized to the internal control GAPDH. Data in (a) represent the mean ± SEM (n=4 independent experiments). Data in (c, d) represent the mean (n= 1 or 2 independent experiments). ns: not significant (unpaired, two-sided Student's *t*-test). Exact *P* values and statistical parameters are provided in Source Data File.

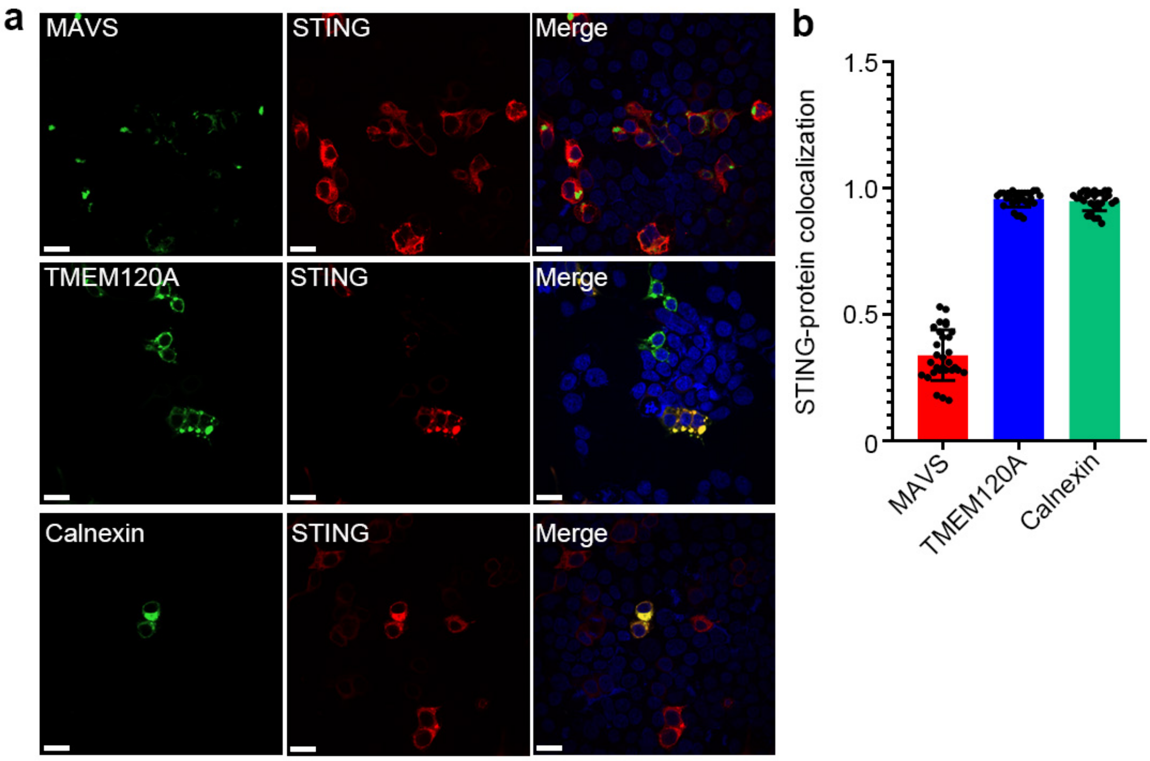

Supplementary Figure 5. TMEM120A co-localizes with STING.

HEK293T cells were transiently transfected with plasmids expressing STING and HA-FLAG tagged MAVS, Calnexin or TMEM120A. 48h post transfection, cells were fixed and permeabilized for immunostaining using STING (red) antibody and FLAG (green) antibody (a). Scale bar, 20 μm. STING-MAVS, Calnexin or TMEM120A co-localization was quantified using Pearson's correlation coefficient method. Cells expressing both STING and MAVS, Calnexin or TMEM120A were selected randomly for co-localization analysis by ImageJ software (b). Calnexin is an ER marker. MAVS is located in the mitochondrial membrane.

Quantification of immunostaining data in (b) represent the mean ± SEM (n=30 cells per group). Exact *P* values and statistical parameters are provided in Source Data File.

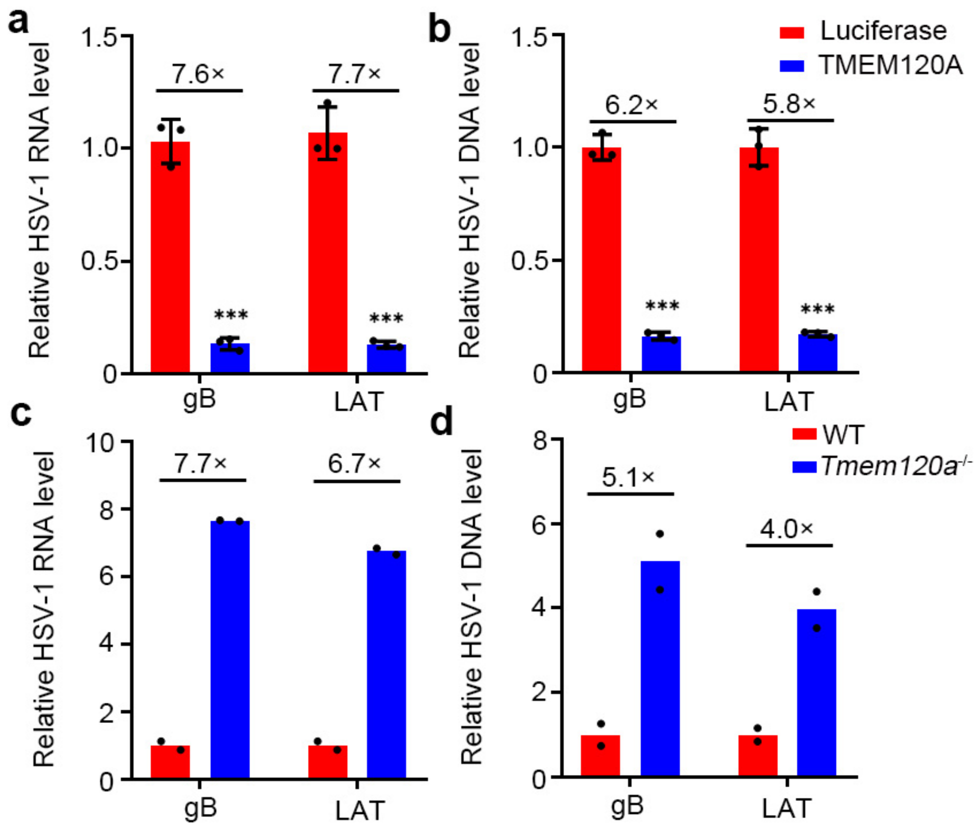

Supplementary Figure 6. TMEM120A inhibits HSV-1 infection.

(a, b) TMEM120A overexpression significantly inhibited HSV-1 infection in U87MG cells. U87MG cells stably expressing HA-FLAG-tagged luciferase or TMEM120A were infected with HSV-1 at an MOI of 0.1 for 24h. Cells were then harvested and primers targeting gB and LAT were utilized to determine the RNA level (a) and DNA (b) of HSV-1 infection.

(c, d) *Tmem120a* deletion significantly promoted HSV-1 infection in MEFs. WT and *Tmem120a*$^{-/-}$ MEFs were infected with HSV-1 at an MOI of 0.2 for 24h. Cells were then harvested and primers targeting gB and LAT were utilized to determine the RNA level (c) and DNA (d) of HSV-1 infection.

Data in (a, b) represent the mean ± SEM (n=3 independent experiments). Data in (c, d) represent the mean (n=2 independent experiments). ***: P<0.001 (unpaired, two-sided Student's t-test). Exact P values and statistical parameters are provided in Source Data File.

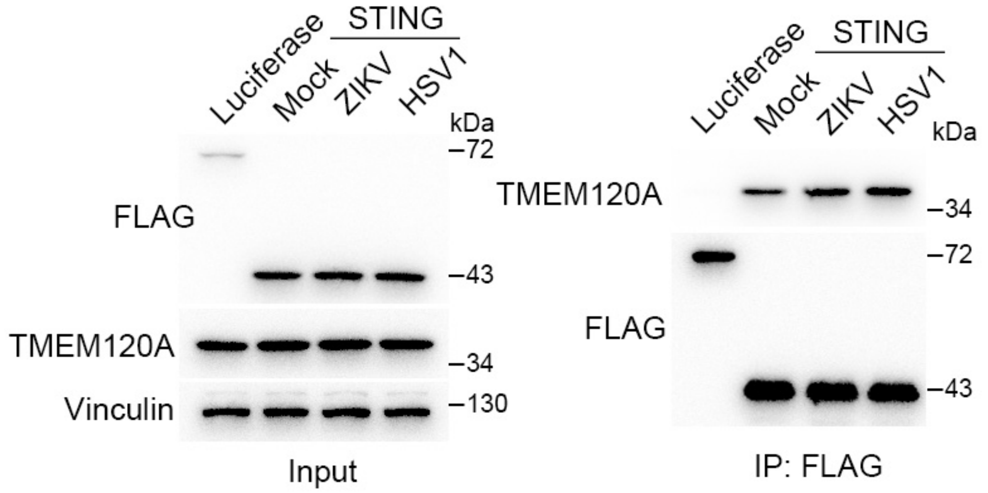

Supplementary Figure 7. ZIKV and HSV-1 infection enhance the association of STING and TMEM120A.

U87MG cells expressing FLAG tagged luciferase or STING and TMEM120A were infected with ZIKV or HSV-1 an MOI of 1 for 12h and then harvested for FLAG-tag based immunoprecipitation and immunoblotting to detect the interaction between TMEM120A and STING or luciferase using TMEM120A antibody. 10% of input was run and blotted.

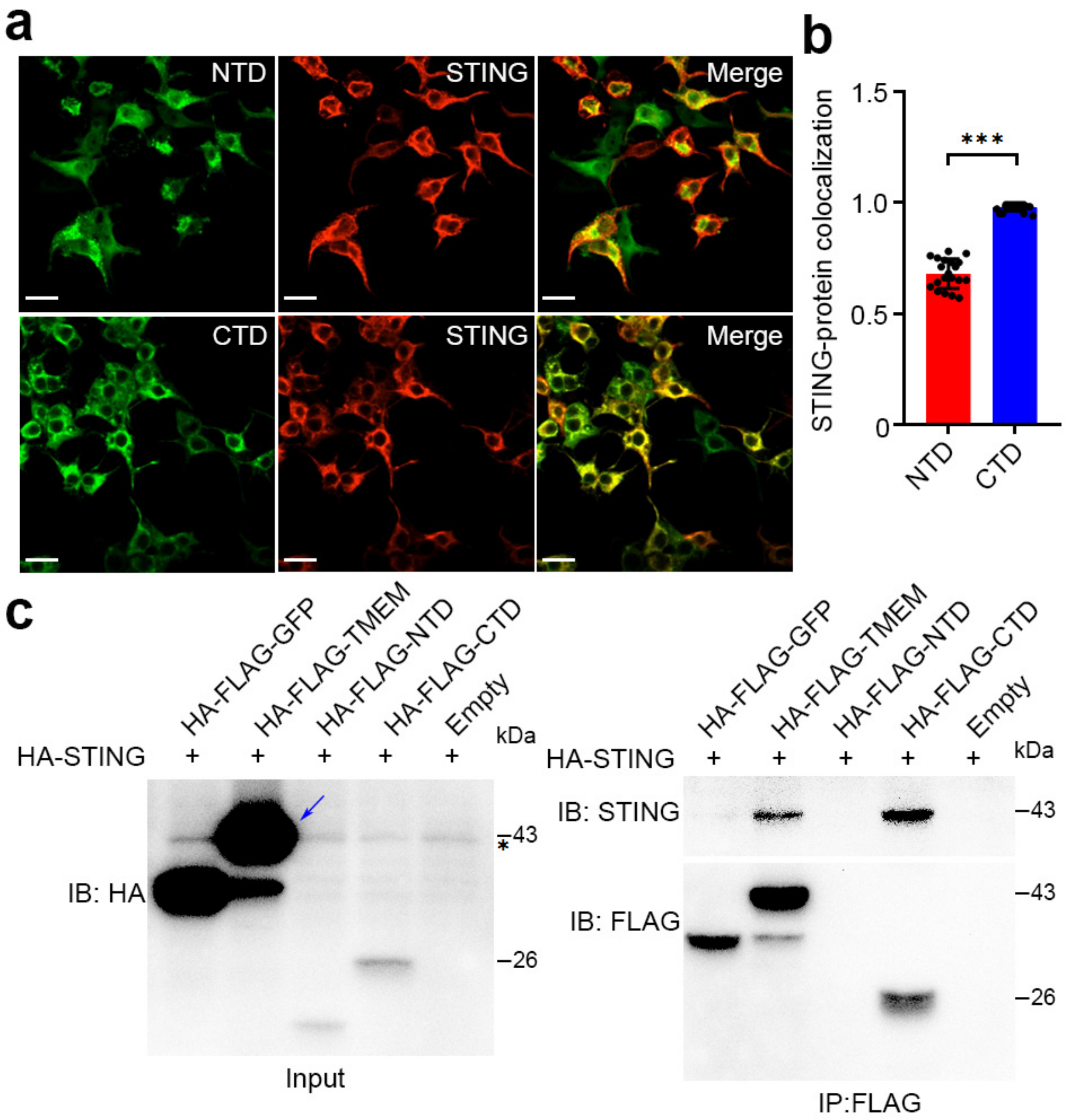

Supplementary Figure 8. The C terminal domain (CTD) of TMEM120A is sufficient for STING binding.

(a-b) TMEM120A CTD co-localized with STING. HEK293T cells were transiently transfected with plasmids expressing STING and HA-FLAG tagged NTD or CTD of TMEM120A. 48h post transfection, cells were fixed and permeabilized for immunostaining using STING (red) and FLAG (green) antibodies and confocal analysis (a). Scale bar, 20 μm. Co-localization of STING and NTD or CTD was quantified using Pearson's correlation coefficient method. Cells expressing both STING and NTD or CTD of TMEM120A were selected randomly for co-localization analysis by ImageJ software (b). ***: P<0.001 (unpaired, two-sided Student's t-test).

(c) The CTD of TMEM120A is sufficient for STING binding. HEK293T cells were co-transfected with plasmids expressing HA-STING and HA-FLAG tagged GFP, TMEM120A, NTD, CTD or empty vector for 48h. Cells were then collected for FLAG-tag based immunoprecipitation and

immunoblotting to detect STING using STING antibody. The sizes of HA-FLAG tagged TMEM120A and HA-STING are similar. 10% of input was run and blotted. Arrow: HA-FLAG tagged TMEM120A. Asterisk: HA-STING.

Quantification of immunostaining data in (b) represent the mean ± SEM (n=20 cells per group). ***: $P<0.001$ (unpaired, two-sided Student's *t*-test). Exact *P* values and statistical parameters are provided in Source Data File.

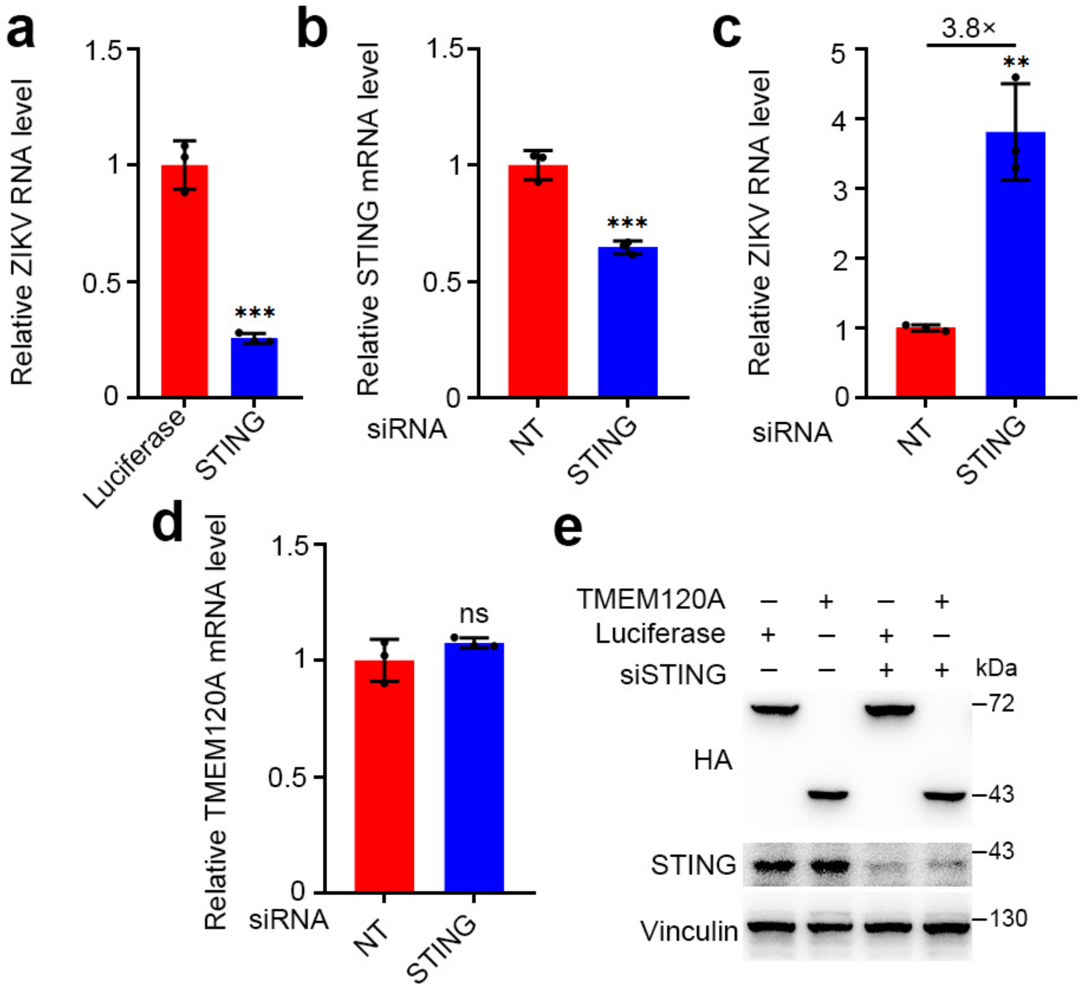

Supplementary Figure 9. STING knockdown does not affect TMEM120A expression.

(a) STING overexpression reduced ZIKV RNA level in U87MG cells. U87MG stably expressing luciferase or STING was infected with ZIKV at an MOI of 0.1 for 48h. Cells were then harvested for RT-qPCR.

(b) Efficiency of siRNA mediated STING knockdown. U87MG cells were transfected with NT siRNA or siRNA targeting STING for 72h and harvested for RT-qPCR to measure STING mRNA level.

(c) siRNA based STING knockdown increased ZIKV RNA level in U87MG cells. U87MG cells were transfected with NT siRNA or siRNA targeting STING for 48h and infected with ZIKV at an MOI of 0.1 for another 48h. Cells were then harvested for RT-qPCR.

(d) siRNA based STING knockdown did not affect TMEM120A mRNA level in U87MG cells. U87MG cells were transfected with NT siRNA or siRNAs targeting STING for 72h and then harvested for RT-qPCR to measure TMEM120A mRNA level.

(e) siRNA based STING knockdown did not affect TMEM120A protein level in U87MG cells. U87MG cells stably expressing HA-FLAG tagged luciferase or TMEM120A were transfected with NT siRNA or siRNAs targeting STING for 72h and then harvested for immunoblotting to measure protein level.

Cellular ZIKV RNA level and gene mRNA level were normalized to the internal control GAPDH. RT-qPCR data represent the mean ± SEM (n=3 independent experiments). **: P<0.01, ***: P<0.001 (unpaired, two-sided Student's t-test). ns: not significant. Exact P values and statistical parameters are provided in Source Data File.

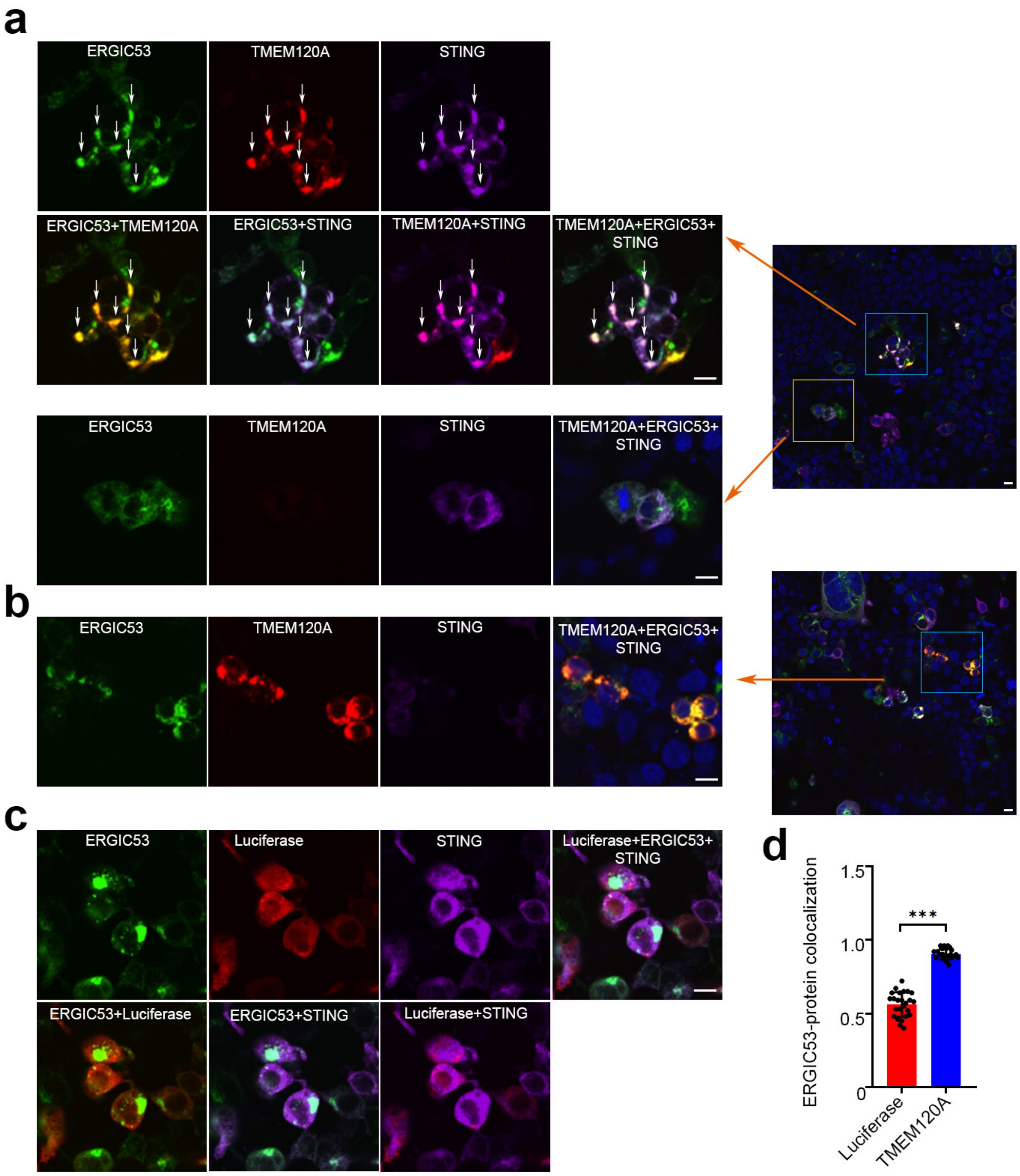

Supplementary Figure 10. TMEM120A co-localizes with STING and ERGIC53.

(a-c) HEK293T cells were transiently transfected with plasmids expressing STING, GFP-ERGIC53 (green) and HA-FLAG tagged luciferase (c) or TMEM120A (a, b). 48h post transfection, cells were fixed and permeabilized for immunostaining using STING (purple) and FLAG (Red) antibodies and confocal analysis. Compared with cells in blue box in (a), cells in yellow box in (a) expressed lower level of TMEM120A and cells in blue box in (b) expressed lower level of STING. Scale bar, 10 μm. White arrows: colocalization foci.

(d) Co-localization of ERGIC53 and TMEM120A or luciferase was quantified using Pearson's correlation coefficient method. Cells expressing both ERGIC53 and TMEM120A or luciferase

were selected randomly for co-localization analysis by ImageJ software. Quantification of immunostaining data in (d) represent the mean ± SEM (n=25 cells per group). ***: $P<0.001$ (unpaired, two-sided Student's *t*-test). Exact *P* values and statistical parameters are provided in Source Data File.

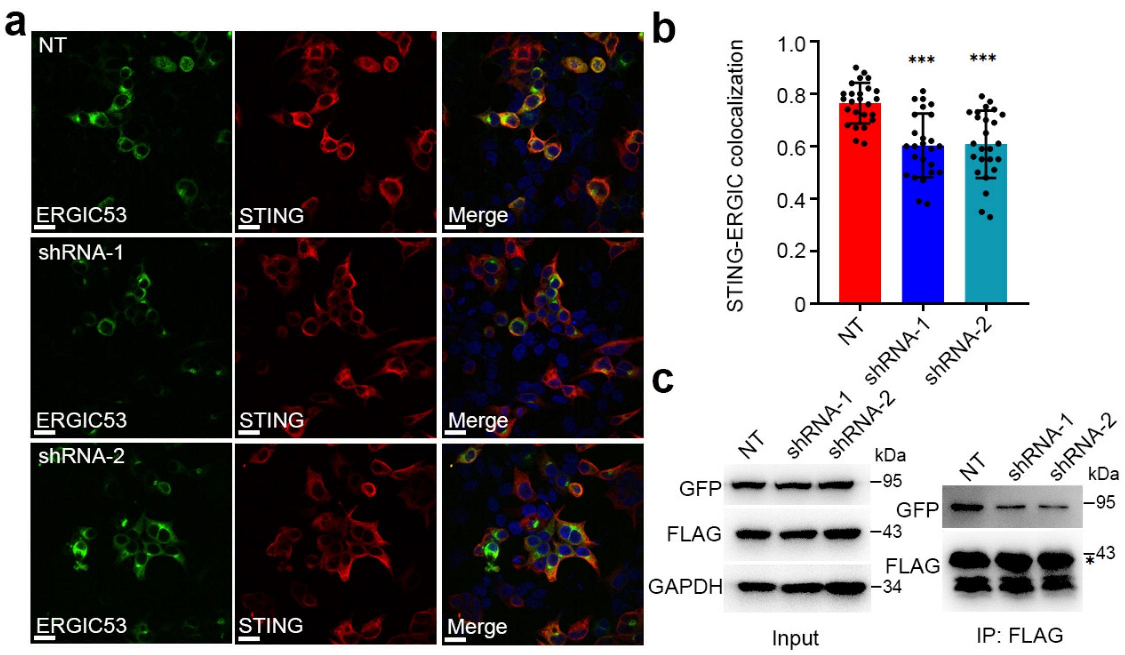

Supplementary Figure 11. TMEM120A knockdown inhibits STING trafficking from the ER to ERGIC.

(a, b) HEK293T cells stably expressing non-targeting control (NT) shRNA or TMEM120A shRNAs were transiently transfected with plasmids expressing STING and GFP-ERGIC53 (green). 48h post transfection, cells were treated with 2 µg/ml cGAMP for 6h, then were fixed and permeabilized for immunostaining using STING (red) antibody followed by confocal analysis (a). Scale bar: 20 µm. STING-ERGIC53 co-localization was quantified using Pearson's correlation coefficient method. Cells expressing both STING and ERGIC53 were selected randomly for co-localization analysis by ImageJ (b). ERGIC53 is an ERGIC marker.

(c) HEK293T cells stably expressing non-targeting control (NT) shRNA or TMEM120A shRNAs were transiently transfected with plasmids expressing STING and GFP-ERGIC53. 48h post transfection, cells were treated with 2 µg/ml cGAMP for 6h, then collected for FLAG-tag based immunoprecipitation and immunoblotting to detect ERGIC53 using GFP antibody. 10% of input was run and blotted. Asterisk: FLAG-STING.

Quantification of immunostaining data in (b) represent the mean ± SEM (n=25 cells per group). ***: P<0.001 (unpaired, two-sided Student's t-test of each shRNA). Exact P values and statistical parameters are provided in Source Data File.

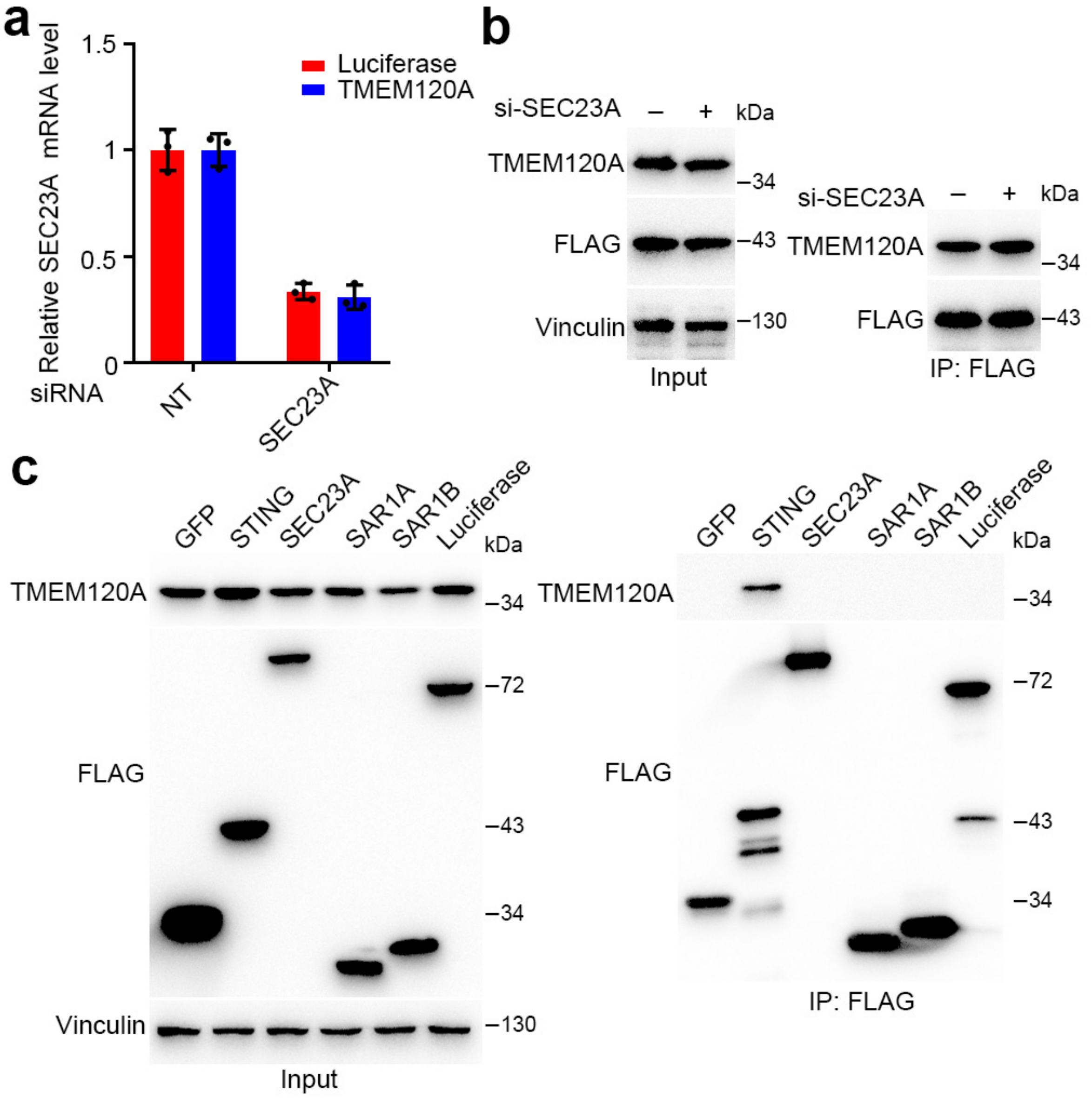

Supplementary Figure 12. siRNA based SEC23A knockdown do not affect the interaction between TMEM120A with STING.

(a) U87MG cells stably expression luciferase or TMEM120A were transfected with NT siRNA or siRNA targeting SEC23A for 72h and harvested for RT-qPCR to measure SEC23A mRNA level. SEC23 mRNA level was normalized to NT group.

(b) SEC23A knockdown did not affect the interaction between TMEM120A with STING. U87MG cells expressing FLAG-STING and TMEM120A were transiently transfected with non-targeting control (NT) siRNA or SEC23A siRNAs. 48h post transfection, cells were then collected for FLAG-tag based immunoprecipitation and immunoblotting to detect the interaction between TMEM120A and STING using TMEM120A antibody. 10% of input was run and blotted.

(c) TMEM120A did not interact with SEC23A, SAR1A or SAR1B. HEK293T cells were transiently transfected with plasmids expressing FLAG-GFP, STING, SEC23A, SAR1A, SAR1B or luciferase. 24h post transfection, cells were transiently transfected with the plasmid expressing

TMEM120A for another 48h. Cells were then collected for FLAG-tag based immunoprecipitation and immunoblotting to detect the interaction using TMEM120A antibody. 10% of input was run and blotted.

RT-qPCR data in (a) represent the mean ± SEM (n=3 independent experiments).

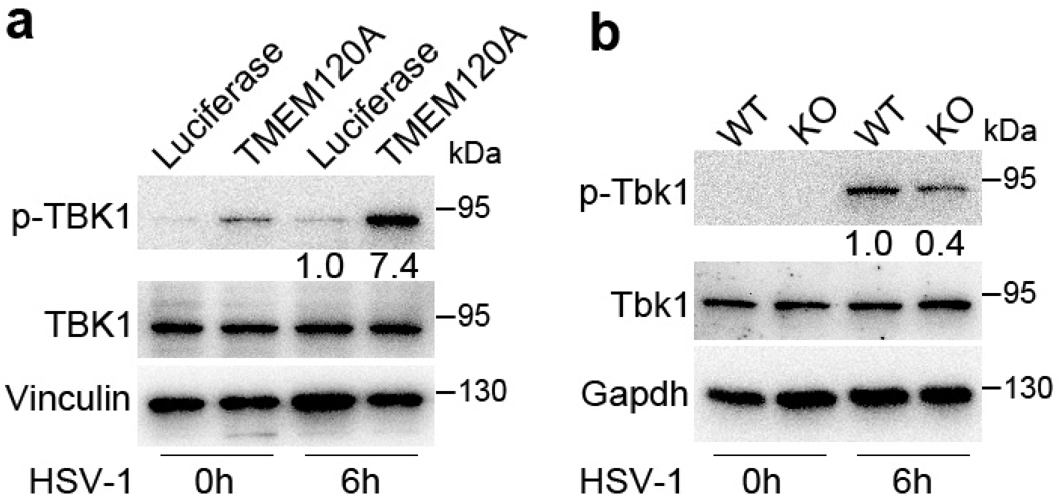

Supplementary Figure 13. TMEM120A mediates HSV-1 induced phosphorylation of TBK1. (a) TMEM120A overexpression increased the phosphorylation of TBK1 after HSV-1 infection in U87MG cells. U87MG cells stably expressing HA-FLAG-tagged luciferase or TMEM120A were infected with HSV-1 at an MOI of 1 for 6h. Cells were collected for immunoblotting to detect the activation of TBK1 using phospho-TBK1 (p-TBK1), TBK1 antibodies. Ratio: p-TBK1/TBK1.
(b) *Tmem120a* deletion reduced the phosphorylated Tbk1 induced by HSV-1 infection in MEFs. WT and *Tmem120a*$^{-/-}$ MEFs were infected with HSV-1 at an MOI of 1 for 6h. Cells were collected for immunoblotting to detect the activation of Tbk1 using p-Tbk1, Tbk1, antibodies. Ratio: p-Tbk1/Tbk1.

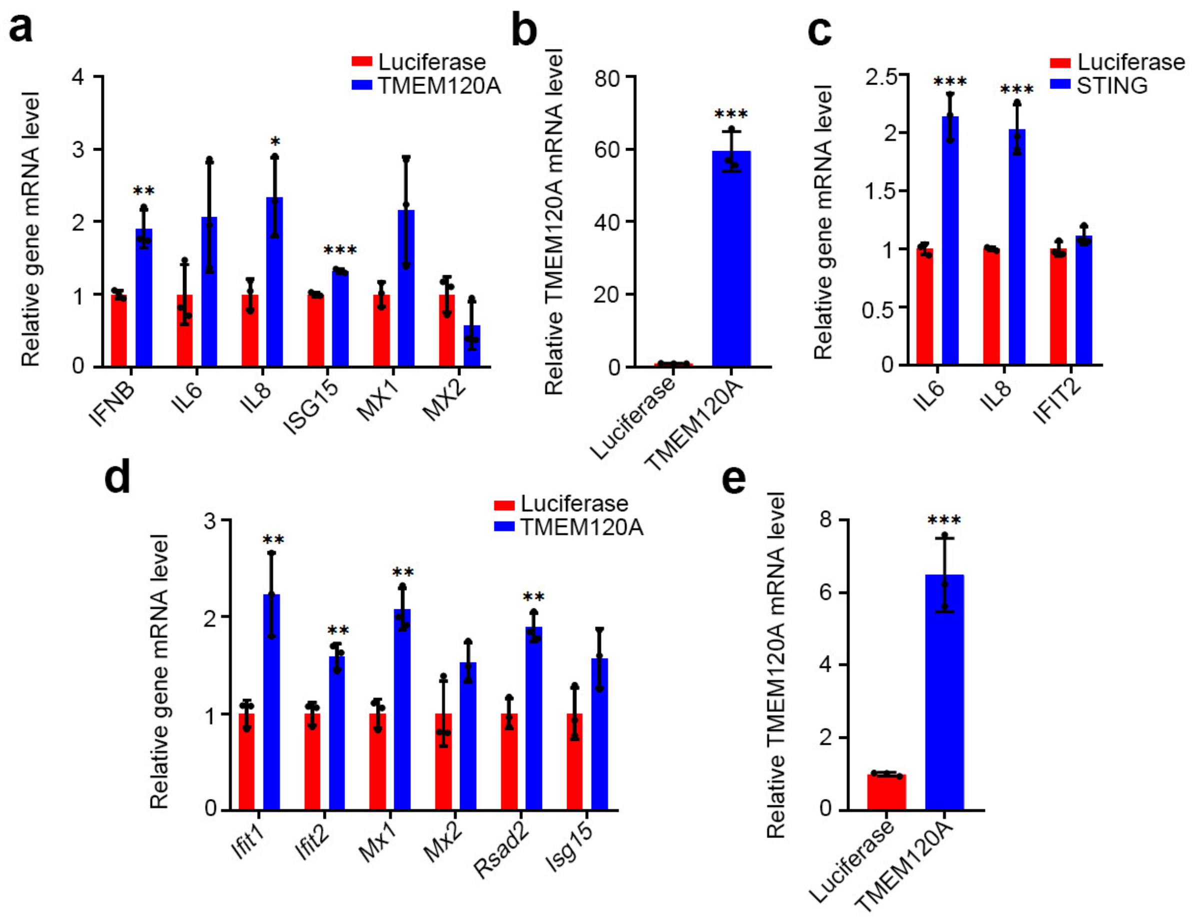

Supplementary Figure 14. TMEM120A promotes the expression of inflammatory cytokines.

(a, b) TMEM120A overexpression promoted type I IFN response in HEK293T cells. HEK293T cells stably expressing STING were transiently transfected with plasmids expressing luciferase or TMEM120A. 40h post transfection, cells were stimulated with 2′3′-cGAMP (2 μg/mL) for 6h. Cells were then harvested for RT-qPCR to detect the mRNA level of IFNB, IL6, IL8, ISG15, MX1, MX2 (a) and TMEM120A (b).

(c) STING overexpression promoted type I IFN response in U87MG cells. U87MG cells stably expressing luciferase or STING were infected with ZIKV at an MOI of 0.1 for 48h. Cells were harvested for RT-qPCR to detect the mRNA level of IL6, IL8 and IFIT2.

(d, e) TMEM120A overexpression promoted type I IFN response in RAW 264.7 cells. RAW 264.7 cells were transiently transfected with plasmids expressing luciferase or human TMEM120A. Cells were harvested for RT-qPCR to detect the mRNA level of *Ifit1*, *Ifit2*, *Mx1*, *Mx2*, *Rsad2*, *Isg15* (d) and TMEM120A (e).

Gene mRNA level was normalized to the internal control GAPDH (*Gapdh*). Data represent the mean ± SEM (n=3 independent experiments). *: P<0.05, **: P<0.01, ***: P<0.001 (unpaired, two-sided Student's t-test). Exact P values and statistical parameters are provided in Source Data File.

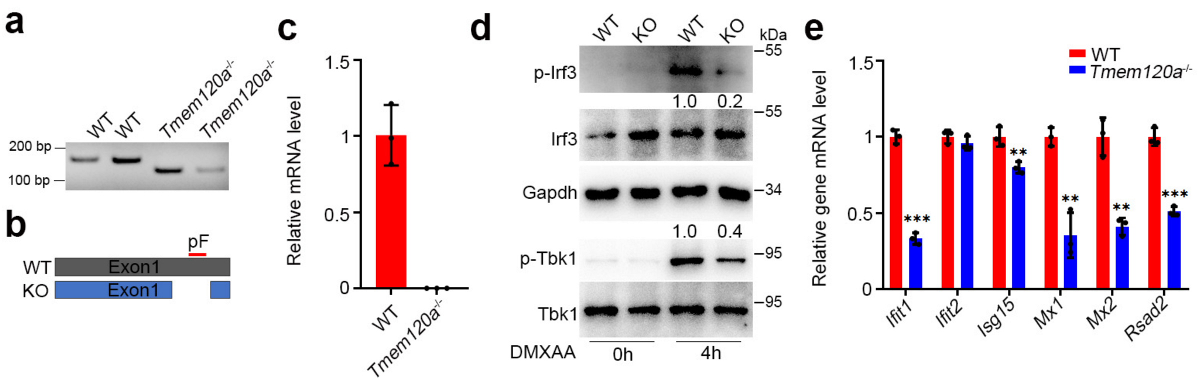

Supplementary Figure 15. *Tmem120a* knockout decreases type I IFN response in MEFs.

(a) Genotyping of mice in Fig.5a.

(b) Schematic of the deletion region by CRISPR-Cas9 gene editing in *Tmem120a*$^{-/-}$ mice. A 77 base-pair (bp) fragment including the start codon ATG was deleted in exon1 of *Tmem120a*. pF, forward primer designed for RT-qPCR.

(c) Confirmation of *Tmem120a* deletion. MEFs were harvested for RT-qPCR to detect *Tmem120a* mRNA level.

(d) *Tmem120a* deletion decreased DMXAA induced Tbk1 and Irf3 phosphorylation in MEFs. WT and *Tmem120a*$^{-/-}$ MEFs were stimulated with DMXAA (10 μg/mL) for the indicated time (0, 4h). Cells were collected for immunoblotting to detect the activation of Tbk1 and Irf3 using p-Tbk1, Tbk1, pIrf3, and Irf3 antibodies. p-Tbk1, Tbk1 blots and p-Irf3, Irf3, Gapdh blots are from two gels with the same samples. Ratio: p-Tbk1/Tbk1 or p-Irf3/Irf3.

(e) *Tmem120a* KO decreases type I IFN response in MEFs. WT and *Tmem120a*$^{-/-}$ MEFs were infected with ZIKV at 1 MOI for 3h. Cells were then harvested for RT-qPCR to detect the mRNA level of *Ifnb1* and other *Isgs*. Cellular ZIKV RNA level and gene mRNA level were normalized to the internal control *Gapdh*. RT-qPCR data in (c, e) represent the mean ± SEM (n=3 independent experiments). **: *P*<0.01, ***: *P*<0.001 (unpaired, two-sided Student's *t*-test). Exact *P* values and statistical parameters are provided in Source Data File.

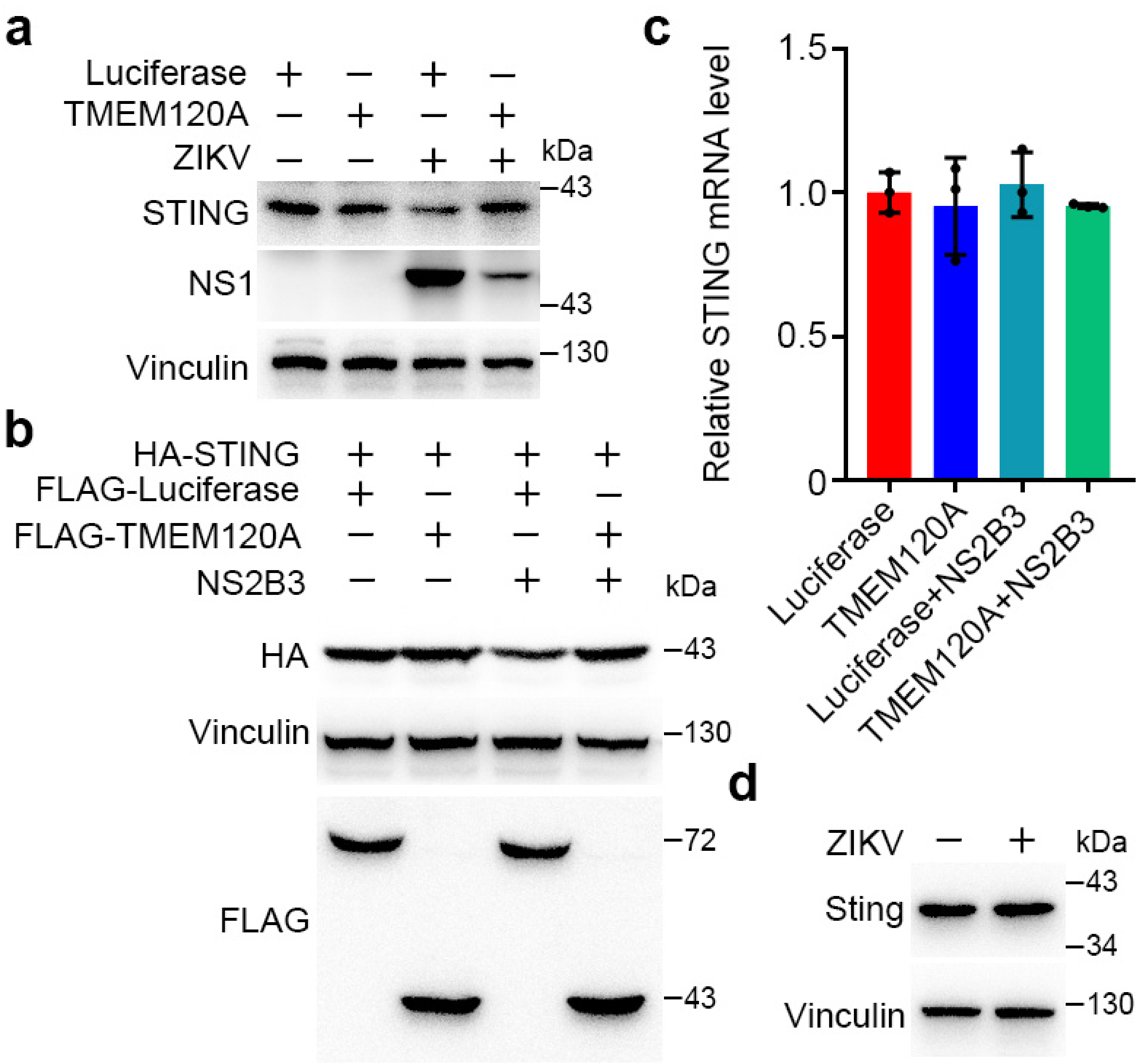

Supplementary Figure 16. TMEM120A overexpression protects STING from cleavage by NS2B3 of ZIKV. (a) TMEM120A overexpression did not increase STING protein level, and ZIKV infection significantly reduced STING protein level in luciferase but not TMEM120A overexpressing U87MG cells. U87MG cells stably expressing HA-FLAG-tagged luciferase or TMEM120A were infected with ZIKV at an MOI of 1 for 48h. Cells were then harvested for immunoblotting (STING antibody).

(b, c) TMEM120A overexpression blocked the reduction of STING protein level induced by NS2B3 expression without affecting STING mRNA level in HEK293T cells. HEK293T cells were transiently transfected with plasmids expressing N-terminal FLAG tagged luciferase or TMEM120A, HA-STING and NS2B3 for 48h. Cells were then collected for immunoblotting (HA antibody) (b) and RT-qPCR (c).

(d) ZIKV infection did not reduce Sting protein level in MEFs. MEFs were infected with ZIKV at an MOI of 1 for 48h. Cells were then harvested for immunoblotting (Sting antibody).

RT-qPCR data in (c) represent the mean ± SEM (n=3 independent experiments).

Supplementary Table 1. Primers for quantitative real time-PCR in this study.

Gene name	Forward (5' to 3')	Reverse (5' to 3')
hGAPDH	GGAGCGAGATCCCTCCAAAAT	GGCTGTTGTCATACTTCTCATGG
DENV	AGTTGTTAGTCTACGTGGACCGA	CGCGTTTCAGCATATTGAAAG
ZIKV	GGTCAGCGTCCTCTCTAATAAACG	GCACCCTAGTGTCCACTTTTTCC
HSV-1-gB	CGCATCAAGACCACCTCCTC	AGCTTGCGGGCCTCGTT
HSV-1-LAT	ACCCACGTACTCCAAGAAGGC	TAAGACCCAAGCATAGAGAGCCA
hSEC23A	GGAGTCCGATTTAGTTGGAATGT	AGGTCTCTCTTTCAGTGGTGT
hISG15	CGCAGATCACCCAGAAGATCG	TTCGTCGCATTTGTCCACCA
hMX1	GTTTCCGAAGTGGACATCGCA	CTGCACAGGTTGTTCTCAGC
hMX2	CAGAGGCAGCGGAATCGTAA	TGAAGCTCTAGCTCGGTGTTC
hIFIT2	AAGCACCTCAAAGGGCAAAAC	TCGGCCCATGTGATAGTAGAC
hIL6	ACTCACCTCTTCAGAACGAATTG	CCATCTTTGGAAGGTTCAGGTTG
hSTING	CACCTGTGTCCTGGAGTACG	AGTGTCCGGCAGAAGAGTTT
hTMEM	CGGGACTGGGAGGATCTACAG	CTTGGCGCTCTTTCATCTGG
hIFNβ	CTTGGATTCCTACAAAGAAGCAGC	TCCTCCTTCTGGAACTGCTGCA
hIL8	TTTTGCCAAGGAGTGCTAAAGA	AACCCTCTGCACCCAGTTTTC
mIfnb	AACCTCACCTACAGGGCGGACTTC	TCCCACGTCAATCTTTCCTCTTGCTTT
mMx1	GACCATAGGGGTCTTGACCAA	AGACTTGCTCTTTCTGAAAAGCC
mMx2	GAGGCTCTTCAGAATGAGCAAA	CTCTGCGGTCAGTCTCTCT
mIrf7	GAGACTGGCTATTGGGGGAG	GACCGAAATGCTTCCAGGG
mIl6	CTGCAAGAGACTTCCATCCAG	AGTGGTATAGACAGGTCTGTTGG
mIfit2	GGAGAGCAATCTGCGACAG	GCTGCCTCATTTAGACCTCTG
mRsad2	AGCATTAGGGTGGCTAGATCC	CTGAGTGCTGTTCCCATCTTC
mIfit1	GCCTATCGCCAAGATTTAGATGA	TTCTGGATTTAACCGGACAGC
mGapdh	AGGTCGGTGTGAACGGATTTG	GGGGTCGTTGATGGCAACA
mIsg15	GGTGTCCGTGACTAACTCCAT	CTGTACCACTAGCATCACTGTG

Supplementary Table 2. STING and SEC23A siRNAs used in this study.

Gene name	Forward (5' to 3')	Reverse (5' to 3')
STING	CCCGGAUUCGAACUUACAATT	UUGUAAGUUCGAAUCCGGGTT
SEC23A-si1	GUUAUGCUGGUAUAUCUGATT	UCAGAUAUACCAGCAUAACTT
SEC23A-si2	GCAUAAUGCUCCAAUUCCUTT	AGGAAUUGGAGCAUUAUGCTT
