# Supplementary material for: Gain-of-function genetic screening identifies the antiviral function of TMEM120A via STING activation
Source: Nat Commun. 2022 Jan 10;13:105. doi: 10.1038/s41467-021-27670-1 (PMC8748537; doi:10.1038/s41467-021-27670-1)
Supplement: Supplementary file 3 — Description of Additional Supplementary Files [file 41467_2021_27670_MOESM3_ESM.pdf]

Description of Additional Supplementary Files

Supplementary Data 1. Raw data and data analysis of human genome-wide ORFs overexpression screen of ZIKV host factors.

Raw results: List of ORF encoded proteins and read counts of ORF barcodes in cells after 10 days non-infection (Mock) or ZIKV infection (duplicate: Exp1 and Exp2). The read counts of ORF were normalized using DEseq2. Exp: experimental group.

Restriction factors: List of restriction factors selected with log2FC > 0.5. log2FC including log2(Exp1/Mock) and log2(Exp1/Mock) means the log2 fold changes (FC) in reads count collected from ZIKV infected cells relative to uninfected cells.

Dependent factors: List of dependent factors selected with log2FC < -0.5. log2FC including log2(Exp1/Mock) and log2(Exp1/Mock) means the log2 fold changes (FC) in reads count collected from ZIKV infected cells relative to uninfected cells.

DE_analysis: Output of differential expression (DE) analysis compared to control. baseMean: mean values from all normalized samples; lfcSE: standard error for log2 fold change; stat: statistic value for the null hypothesis; padj: false discovery rate.
