# Supplementary material for: Gain-of-function genetic screening identifies the antiviral function of TMEM120A via STING activation
Source: Nat Commun. 2022 Jan 10;13:105. doi: 10.1038/s41467-021-27670-1 (PMC8748537; doi:10.1038/s41467-021-27670-1)
Supplement: Supplementary file 5 — Reporting Summary [file 41467_2021_27670_MOESM5_ESM.pdf]

nature portfolio

Corresponding author(s): Xu Tan

Last updated by author(s): Nov 5, 2021

Reporting Summary

Nature Portfolio wishes to improve the reproducibility of the work that we publish. This form provides structure for consistency and transparency in reporting. For further information on Nature Portfolio policies, see our Editorial Policies and the Editorial Policy Checklist.

Statistics

For all statistical analyses, confirm that the following items are present in the figure legend, table legend, main text, or Methods section.

n/a	Confirmed	
☐	☒	The exact sample size (n) for each experimental group/condition, given as a discrete number and unit of measurement
☐	☒	A statement on whether measurements were taken from distinct samples or whether the same sample was measured repeatedly
☐	☒	The statistical test(s) used AND whether they are one- or two-sided<br>*Only common tests should be described solely by name; describe more complex techniques in the Methods section.*
☒	☐	A description of all covariates tested
☒	☐	A description of any assumptions or corrections, such as tests of normality and adjustment for multiple comparisons
☐	☒	A full description of the statistical parameters including central tendency (e.g. means) or other basic estimates (e.g. regression coefficient) AND variation (e.g. standard deviation) or associated estimates of uncertainty (e.g. confidence intervals)
☐	☒	For null hypothesis testing, the test statistic (e.g. F, t, r) with confidence intervals, effect sizes, degrees of freedom and P value noted<br>*Give P values as exact values whenever suitable.*
☒	☐	For Bayesian analysis, information on the choice of priors and Markov chain Monte Carlo settings
☒	☐	For hierarchical and complex designs, identification of the appropriate level for tests and full reporting of outcomes
☐	☒	Estimates of effect sizes (e.g. Cohen's d, Pearson's r), indicating how they were calculated

Our web collection on statistics for biologists contains articles on many of the points above.

Software and code

Policy information about availability of computer code

Data collection	HiSeq2000(Illumina)<br>Bio-Rad CFX96 Real-Time System<br>Thermo ARRAYSCAN VTI HCS Reader<br>Olympus FV1000<br>Multimode plate reader (PerkinElmer)<br>Multimode plate reader (PerkinElmer)<br>spinning disc confocal microscope (PerkinElmer or Andor)
Data analysis	Bio-Rad CFX Maestro<br>ImageJ-win64(Fiji.app)<br>GraphPad Prism 8.0<br>Python2.7<br>R 3.5<br>Imaris9<br>Cutadapt (version 1.18)<br>Samtools (version 1.3.1)<br>Bowtie2-2.2.5<br>DEseq2<br>Excel 2016

For manuscripts utilizing custom algorithms or software that are central to the research but not yet described in published literature, software must be made available to editors and reviewers. We strongly encourage code deposition in a community repository (e.g. GitHub). See the Nature Portfolio guidelines for submitting code & software for further information.

Data

Policy information about availability of data

All manuscripts must include a data availability statement. This statement should provide the following information, where applicable:

- Accession codes, unique identifiers, or web links for publicly available datasets
- A description of any restrictions on data availability
- For clinical datasets or third party data, please ensure that the statement adheres to our policy

All data that support the findings of this study are provided in the main Article, Supplementary Information and Source Data File. Any other relevant information can be obtained from the corresponding authors upon reasonable request. Source data are provided with this paper.

Field-specific reporting

Please select the one below that is the best fit for your research. If you are not sure, read the appropriate sections before making your selection.

- [x] Life sciences
- [ ] Behavioural & social sciences
- [ ] Ecological, evolutionary & environmental sciences

For a reference copy of the document with all sections, see nature.com/documents/nr-reporting-summary-flat.pdf

Life sciences study design

All studies must disclose on these points even when the disclosure is negative.

Sample size	No statistical methods were used to predetermine sample size. All experiments with statistical analysis were performed at least three independent times unless otherwise stated, each with multiple technical replicates.
Data exclusions	No data were excluded.
Replication	As mentioned with all relevant figures, sufficient technical and biological replicates of the experiments were performed and the data is reproducible using the methods and models described by the authors. All experiments were replicated at least twice unless otherwise stated.
Randomization	For the immunostaining studies, cells were randomly selected for co-localization analysis.
Blinding	No blinding was performed in the study. All data were acquired and analyzed automatically using the software described above.

Reporting for specific materials, systems and methods

We require information from authors about some types of materials, experimental systems and methods used in many studies. Here, indicate whether each material, system or method listed is relevant to your study. If you are not sure if a list item applies to your research, read the appropriate section before selecting a response.

Materials & experimental systems

n/a	Involved in the study	
[ ]	[x]	Antibodies
[ ]	[x]	Eukaryotic cell lines
[x]	[ ]	Palaeontology and archaeology
[ ]	[x]	Animals and other organisms
[x]	[ ]	Human research participants
[x]	[ ]	Clinical data
[x]	[ ]	Dual use research of concern

Methods

n/a	Involved in the study	
[x]	[ ]	ChIP-seq
[x]	[ ]	Flow cytometry
[x]	[ ]	MRI-based neuroimaging

Antibodies

Antibodies used	The information of all antibodies used are described in the manuscript, including anti-HA (Mouse, BioLegend, MMS-101R-200, 1:2000), anti-TMEM120A (Rabbit, Proteintech, 17455-1-AP, 1:100), anti-STING (Rabbit, Proteintech,19851-1-AP, 1:1000), anti-STING (D2P2F, Rabbit, Cell Signaling Technologies 13647S, 1:1000), anti-p-IRF3 (4D4G, Rabbit, Cell Signaling Technologies, 4947, 1:500), anti-p-TBK1 (D52C2, Rabbit, Cell SignalingTechnologies, 5483, 1:1000), anti-IRF3 (Rabbit, Cell Signaling Technologies, 4302, 1:1000), anti-TBK1 (Rabbit, Cell Signaling Technologies, 3504, 1:1000), anti-GFP (Mouse, Abgent, AM1009a, 1:1000), anti-GAPDH (Mouse, ZSGB-Bio, TA-08, 1:1000), anti-Vinculin (Mouse, Sigma, V4505, 1:5000), anti-Flag (Mouse, Sigma, F1084, 1:2000), anti-Calnexin (Rabbit, Abcam, ab22595, 1:100),peroxidase-conjugated AffiniPure anti-rabbit antibody (ZSGB-Bio Cat#ZB-2301; RRID:AB_2747412,1:10000) ; peroxidase-conjugated AffiniPure anti-mouse antibody (ZSGB-Bio Cat#ZB-2305; RRID:AB_2747415,1:10000).Goat-anti-Mouse Alexa Fluor 488 (Invitrogen, A-11001, 1:1000), Goat-anti-Rabbit Alexa Fluor 568 (Invitrogen, A-11010, 1:1000), Goat-anti-mouse Alexa Fluor 594 (Invitrogen, A-11005, 1:1000), Donkey-anti-Rabbit Alexa Fluor 647

	(Invitrogen, A-31573, 1:1000).
Validation	Most of the antibodies have been pre-validated by the manufacturer and previously reported in the literature. All antibodies were validated in the lab by band specificity and product size in western blot experiments. Antibodies for TMEM120A and STING were further validated for quantitative detection of decreases in protein expression following knock-down with gene-specific siRNAs and/or shRNAs

Eukaryotic cell lines

Policy information about cell lines

Cell line source(s)	HEK293T, U87MG, C6 and Vero E6 and RAW264.7 cells were from ATCC.<br>Huh7 cells were provided by Dr. Wenhui Li, National Institute of Biological Sciences, Beijing (from the Cell Bank of Type Culture Collection, Chinese Academy of Sciences).
Authentication	Cells were checked permanently according to morphology and function features or resistant to certain antibiotics
Mycoplasma contamination	Cell lines have been regularly test for mycoplasma contamination using a commercial kit (Vazyme). If contaminated, cells would be discarded.
Commonly misidentified lines<br>(See ICLAC register)	No commonly misidentified cell lines were used.

Animals and other organisms

Policy information about studies involving animals; ARRIVE guidelines recommended for reporting animal research

Laboratory animals	12 weeks C57BL/6 female mice were used as embryo donors and pseudo-pregnant foster mothers for the generation of TMEM120A knockout mouse. Embryonic day (E) 14.5 mouse embryos of both sexes were collected for the isolation of mouse embryonic fibroblasts (MEFs).
Wild animals	No wild animals were used in this study
Field-collected samples	No field-collected samples were used in this study.
Ethics oversight	All animal experiments in this study were approved by the Institutional Animal Care and Use Committee (IACUC) at Tsinghua University (approval number: 17TX-1). All mice were housed under specific-pathogen free conditions.

Note that full information on the approval of the study protocol must also be provided in the manuscript.
